# A robust phylogenetic framework for ophiostomatoid fungi: orders *Ophiostomatales* and *Microascales* (*Sordariomycetes*, *Ascomycota*)

**DOI:** 10.3897/mycokeys.140.201772

**Published:** 2026-09-11

**Authors:** Theo Llewellyn, Alfried P. Vogler

**Affiliations:** 1 Leverhulme Centre for the Holobiont, Department of Life Sciences, Imperial College London, Silwood Park Campus, Ascot, Berkshire, SL5 7PY, UK Leverhulme Centre for the Holobiont, Department of Life Sciences, Imperial College London Berkshire United Kingdom; 2 Department of Life Sciences, Natural History Museum, Cromwell Road, London, SW6 7BD, UK Department of Life Sciences, Natural History Museum London United Kingdom

**Keywords:** Fungal lifestyle, insect symbiont, metabarcoding, molecular markers, plant pathogen, systematics

## Abstract

Ophiostomatoids are an ecological group of microfungi that commonly associate with bark and ambrosia beetles. In addition to being insect symbionts, they play significant ecological roles as plant pathogens and include species responsible for major forest tree diseases. Despite their ecological similarities, ophiostomatoid fungi are distributed across two distantly related orders, *Microascales* and *Ophiostomatales*, which include many additional fungi with diverse ecological lifestyles. Previous studies have typically focused on localised taxonomic issues or specific fungal ecologies. As a result, public repositories suffer from fragmented locus coverage, sequence misidentifications, and uneven taxon sampling, leaving the community without a standardised, comprehensive dataset to study these fungi at scale. To address this, all available ophiostomatoid fungal sequence data across seven widely used marker loci were curated and analysed to reconstruct densely sampled alignments and trees covering 918 concatenated *Microascales* sequences and 859 *Ophiostomatales* sequences. Evaluation of locus performance showed that whilst individual loci exhibit extreme state frequency heterogeneity, composition bias, and topological discordance, concatenated datasets produce robust, well-supported topologies consistent with published genomic studies. Integrating phylogenies with broad ecological trait data reveals a clear contrast in data distribution between the two orders, with ecological lifestyles and beetle associations being broadly uniform and stable across the *Ophiostomatales* tree but highly heterogeneous in the *Microascales*, despite comparable species richness. These macroecological data distributions are also shaped by taxonomic philosophies, whereby the fragmentation of *Microascales* into small, restricted genera tends to visually amplify trait variation, whereas the large, heterogeneous genera of *Ophiostomatales* can obscure it. This unified, fully reproducible framework establishes the stable topology and alignments necessary for future molecular taxonomy and trait evolution analyses, while providing a high-quality reference for environmental sequence identification, which in turn can support future biodiversity and biosecurity research.

## Introduction

Ophiostomatoids are a polyphyletic group of fungi from the orders *Ophiostomatales* and *Microascales* (*Sordariomycetes*, *Ascomycota*). Despite being distantly related within the *Sordariomycetes* (Hausner et al. 1993a, 1993b; Spatafora and Blackwell 1994; Li et al. 2021), these fungi share key morphological traits related to insect dispersal, such as flask-shaped ascomata and sticky spore droplets (Luttrell 1951; Benny and Kimbrough 1980; von Arx and van der Walt 1988). They are commonly associated with bark and ambrosia beetles (*Scolytinae* and *Platypodinae*, *Curculionidae*), forming a range of symbiotic interactions from facultative and commensal dispersal to obligate reciprocal mutualism (Hulcr and Stelinski 2017; Biedermann and Vega 2020). The best known of these cause major plant diseases such as Dutch elm disease and laurel wilt (Brasier 1991; Harrington et al. 2008). Some associations also represent rare cases of beetle-mediated “fungal agriculture”, in which beetle hosts farm fungal cultivars as a food source (Farrell et al. 2001).

Although ophiostomatoid fungi are distributed across two distinct orders, their shared morphology led taxonomists to consider them a single natural group for many years (Nannfeldt 1932; Luttrell 1951). Only in 1980 did Benny and Kimbrough (1980) recognise the *Microascaceae* and *Ophiostomataceae* as members of different orders based on conidial and ascospore structure, describing the new order *Ophiostomatales* and redefining the *Microascales* to contain the *Microascaceae*, *Chadefaudilleaceae*, and *Pithoascaceae*. At that time, the *Ophiostomataceae* contained four genera: *Ophiostoma*, *Ceratocystiopsis*, *Sphaeronaemella*, and *Ceratocystis*. Shortly after, Upadhyay (1981) synonymised *Ophiostoma*, *Sphaeronaemella*, *Grosmannia*, and *Europhium* with *Ceratocystis* based on similar long-necked ascocarps producing sticky, sheathed spores, making *Ceratocystis* the type genus of *Ophiostomataceae*.

Subsequent molecular studies revealed that the *Ophiostomatales* genera *Ophiostoma* and *Ceratocystis* were, in fact, distantly related, and *Ceratocystis* was moved out of the *Ophiostomatales* and back into the *Microascales* (Hausner et al. 1993a, 1993b, 1993c; Spatafora and Blackwell 1994). Since then, both orders have undergone considerable taxonomic revision. Most changes in the *Ophiostomatales* have focused on genus descriptions and delimitations (Zipfel et al. 2006; De Beer et al. 2013; de Beer et al. 2016a, 2016b; van der Linde et al. 2016; Bateman et al. 2017; Poinar and Vega 2018; Nel et al. 2021). Recently, de Beer et al. (2022) produced a full genus-level revision for the *Ophiostomatales* based on sequences from four loci and over 200 species, supported by a genome-level phylogenetic backbone. Similarly, the *Microascales* has undergone many genus-level revisions (De Beer et al. 2013; de Beer et al. 2014; Mayers et al. 2015; Nel et al. 2018; Mayers et al. 2020) as well as significant changes at the family level, expanding from four families to eight in the past two decades (Cannon and Kirk 2007; Kirk et al. 2008; Réblová et al. 2011; Wijayawardene et al. 2022; Bao et al. 2023).

Both orders have unresolved phylogenetic issues, including whether to split the single *Ophiostomatales* family *Ophiostomataceae* into smaller, more morphologically consistent families (de Beer et al. 2022) and whether the *Microascales* genera *Tubakiella* and *Nautosphaeria* could represent a new family for the order (Sakayaroj et al. 2011; Hyde et al. 2020; Bao et al. 2023). Additionally, the placement of many genera remains uncertain in both the *Microascales* (De Beer et al. 2013; Bao et al. 2023) and *Ophiostomatales*, despite the use of genomic data (Vanderpool et al. 2018; Nel et al. 2021).

Although *Microascales* and *Ophiostomatales* are best known for their beetle associations, both orders contain a considerable diversity of other ecological lifestyles, from wood-decaying saprotrophs specialised on marine driftwood (*Halosphaeriaceae*, *Microascales*) to globally distributed human pathogens such as *Sporothrix schenckii* (*Ophiostomatales*) (López-Romero et al. 2011; Sakayaroj et al. 2011). This suggests that beetle association is highly homoplastic across ophiostomatoid fungi, highlighting them as an intriguing case study of convergent lifestyle evolution that can be explored in greater detail using robust phylogenies.

To resolve taxonomic issues and explore the evolution of key traits such as trophic modes and symbiotic interactions, a robust, well-sampled phylogenetic framework is needed. However, existing datasets suffer from uneven taxon coverage due to habitat- or lifestyle-specific sampling, and recent environmental metabarcoding has flooded public datasets with unidentified or mislabelled operational taxonomic units (OTUs). This study, therefore, aimed to standardise and consolidate the highly fragmented, often confusingly intertwined literature and sequence datasets into a unified, accessible community resource to support future work on ophiostomatoid fungal biodiversity and evolutionary ecology. All publicly available sequence data for the *Microascales* and *Ophiostomatales* across seven widely used fungal nuclear markers were compiled to reconstruct densely sampled reference phylogenies for each order based on named, vouchered samples. The properties of different loci and their performance in supporting higher taxa were tested, and the resulting trees were coupled with ecological lifestyle and beetle association data to explore patterns of lifestyle diversity both within and between orders.

## Methods

### Sequence data curation and taxon sampling

Sequence data for representatives of the *Microascales* and *Ophiostomatales*, along with their closest outgroups, were compiled based on the genome tree of the kingdom *Fungi* (Li et al. 2021) and the reference phylogeny of JGI Mycocosm (Grigoriev et al. 2014). For *Ophiostomatales*, *Magnaporthales* was used as the outgroup, and for *Microascales*, *Torpedosporales*, *Coronophorales*, and *Glomerellales* were used. Seven gene regions were targeted: three from the nuclear rRNA operon gene cluster, namely the internal transcribed spacer (covering ITS1, 5.8S, and ITS2), the small subunit (SSU/18S), and the large subunit (LSU/28S) rDNA, as well as the protein-coding loci β-tubulin, elongation factor 1α (EF1α), DNA-directed RNA polymerase II largest subunit gene (RPB1), and DNA-directed RNA polymerase II second-largest subunit gene (RPB2).

All subsequent steps were performed separately for *Microascales* and *Ophiostomatales*. For ingroup sampling, all sequences in the NCBI Nucleotide database under the taxon IDs for *Microascales* (taxid:5592) and *Ophiostomatales* (taxid:5151) were downloaded for each gene individually. All potential variants of gene names were included in the search strings. Protein-coding genes were capped at 6000 bp to avoid genomic data, and rRNA operon sequences were filtered to remove environmental samples and uncultured or unidentified sequences. Representative outgroup sequences were downloaded from the six-gene *Fungi* reference set v3 in the Tree-Based Alignment Selector (T-BAS) toolkit (Carbone et al. 2019).

As ITS, SSU, and LSU regions are often sequenced and uploaded as a single DNA fragment, ITSx (Bengtsson-Palme et al. 2013) was used to split and sort all rRNA sequences into the three separate loci, using a minimum sequence length of 100 bp for SSU and LSU. This minimum sequence length was set to avoid including very short flanking regions sequenced in studies that focused on ITS, which would otherwise be split off by ITSx.

### Sequence processing and alignment

Sequences for each gene were aligned using MAFFT v. 7 with the ‘--auto’ strategy (Katoh 2002). β-tubulin paralogue (tubC) genes in the *Ophiostomatales* were also checked and removed by comparing sequences with those identified by Wang et al. (2020). For many taxa in *Microascales*, two β-tubulin copies (β-tubulin 1 and β-tubulin 2) were observed, as discussed by Zhao et al. (2014) and Tekpinar and Kalmer (2019). These were split and aligned separately based on FASTA header information and initial full alignments using MAFFT, resulting in an eight-locus dataset for *Microascales*.

Alignments were manually inspected and curated in AliView, and any sequences from non-ophiostomatoid taxa were removed. Raw FASTA headers were then standardised to TAXON_NAME_STRAIN using command-line sed functions to ensure consistency across loci. Poorly aligned sequences were identified in each locus alignment, and Basic Local Alignment Search Tool (BLAST) searches (Camacho et al. 2009) against the NCBI nt database were used to identify and remove any misidentified or contaminant sequences. After cleaning the alignments, gaps were removed, and the sequences were realigned.

For each locus, MEGA11 (Stecher et al. 2020; Tamura et al. 2021) was used to calculate the GC%, AT skew, and average pairwise distance using the Kimura 2-parameter (K2P) model and 100 bootstrap replicates (Kimura 1980). For K2P distances, all ambiguous positions were removed for each sequence pair (pairwise deletion option). Additionally, chi-square tests of homogeneity of base frequencies were performed using the command-line implementation of PAUP v. 4.0a to determine whether base frequencies differed across taxa in each alignment (Swofford 2003).

### Phylogenetic tree inference

Individual locus alignments were concatenated using AMAS (Borowiec 2016). Different loci from the same strain were identified using strain IDs or known fungal culture collection accession codes, such as CBS accessions in the FASTA headers. For ambiguous cases, the original publications were checked to link sequence IDs to strain IDs. Using both strain IDs and culture codes allowed sequences from different studies that used the same original sample material to be connected. Partitioning schemes were generated in AMAS. Outgroup sequences were added to ingroup alignments using MAFFT’s ‘--addfragments’ function to avoid splitting shorter sequences across full-length alignments.

For each taxon, all concatenated sequences with unique gene combinations were retained using a custom Python script (sequence_selector.py). When a single taxon had multiple concatenated sequences with the same gene combination, the longest sequence was retained. Where possible, concatenated sequences containing the universal fungal barcode region ITS were also retained to make the trees and alignments more usable for future studies using phylogenetic placement.

Maximum likelihood (ML) phylogenetic tree searches were performed with IQ-TREE v. 3 (Minh et al. 2020). For concatenated species-tree analyses, ModelFinder was used with each locus in a separate partition but allowing for partition merging (-m MFP+MERGE) (Kalyaanamoorthy et al. 2017). Nodal support was evaluated using SH-aLRT (1,000 replicates) and ultrafast bootstrap (1,000 replicates). Individual gene trees were also estimated with IQ-TREE using the partitioned dataset (Chernomor et al. 2016; Hoang et al. 2018) with node support assessed via 1,000 ultrafast bootstrap replicates. Using IQ-TREE, gene concordance factors (gCF), which measure the proportion of decisive gene trees supporting each branch in the concatenated species tree, and site concordance factors (sCF), which measure the proportion of decisive sites in the concatenated alignment that support each species branch, were also calculated (Minh et al. 2020). For each gene, the normalised quartet support was also calculated using ASTRAL-III (Zhang et al. 2018). This represents the proportion of quartets in each gene tree that are observed in the species tree.

Long branches or anomalous placements suggestive of misidentification, misalignment, or sequencing artefacts were identified. Sequences flagged as problematic were subjected to BLAST searches to verify their identification and were either realigned where possible or removed from the dataset. Alignments and trees were reanalysed iteratively until no clear anomalies remained. For the final trees, the taxonomic retention index (tRI) was calculated to determine the level of taxonomic monophyly using the phylofuncs.R script from the phylostuff repository (https://github.com/tjcreedy/phylostuff).

### Fungal lifestyle data curation

Given the diversity of fungal lifestyles in the *Ophiostomatales* and *Microascales*, fungal lifestyle data from the FUNGuild database (Nguyen et al. 2016) were mapped onto the concatenated ML phylogenies to observe how lifestyles are distributed across the trees. Lifestyles were assigned using the FUNGuildR package and a custom loop to first assign guilds at the species level and, when species were not present, at the genus and then family levels. A literature search was also performed to identify which genera or species complexes are associated with bark and ambrosia beetles.

All bioinformatic code used to download and process sequences, build trees, and calculate summary statistics can be found at https://github.com/theo-llewellyn/Ophiostomatoid_Trees.

## Results

### Benchmarking locus performance for molecular systematics

A total of 18,121 *Microascales* sequences were downloaded. After standardising names, identifying misidentified sequences, and filtering by species name, 1,716 remained. Concatenating loci resulted in a final *Microascales* alignment with 918 concatenated sequences and 15,995 sites, of which 5,453 were informative. The final alignment covered all *Microascales* families except two small ones, *Cornuvesicaceae* and *Triadelphiaceae*. For the *Ophiostomatales*, 17,147 sequences were downloaded. After standardising names and identifying misidentified sequences, 1,947 remained. Concatenating loci resulted in a final *Ophiostomatales* alignment with 859 concatenated sequences and 21,603 sites, of which 4,363 were informative. Suppl. material 1 shows the taxa in each concatenated alignment and the NCBI accession codes for each sequence. Suppl. material 1 also shows details of misidentified sequences and the reasons for their exclusion based on BLAST or literature searches.

To evaluate the contribution of individual loci to phylogenetic inference, locus alignment statistics and gene-wise clade support were compared against the concatenated species tree. The number of available sequences varied widely among loci in both orders. For the *Microascales*, ITS had the most sequences, at 751, compared with only 40 for RPB2 (Table 1). The number of informative sites was highest for ITS (1,132/2,673) and lowest for SSU (269/2,239). For *Ophiostomatales*, ITS also had the highest number of sequences, whilst RPB1 had the lowest (634 and 9, respectively), and β-tubulin had both the longest alignment and the most informative sites (Table 1).

**Table 1.** Alignment summary statistics for the *Microascales* eight-locus and *Ophiostomatales* seven-locus concatenated alignments and individual locus alignments.

**Dataset**	**Locus**	**Sequences**	**Sites**	**Informative**	**Invariant**
* Microascales *	Concatenated	918	15995	5453	8896
	SSU	75	2239	269	1804
	ITS	751	2673	1132	1198
	LSU	138	2583	450	1824
	β-tubulin 1	158	839	281	465
	β-tubulin 2	254	2274	1055	1494
	EF1α	255	2514	1101	1132
	RPB1	45	1260	608	532
	RPB2	40	1113	557	447
* Ophiostomatales *	Concatenated	859	21603	4363	15069
	SSU	28	2811	110	2540
	ITS	634	2885	943	1687
	LSU	315	3452	253	2972
	β-tubulin	505	3504	1405	1464
	EF1α	296	3002	998	1735
	RPB1	9	3146	128	2503
	RPB2	160	2803	526	2168

**Figure 1.**
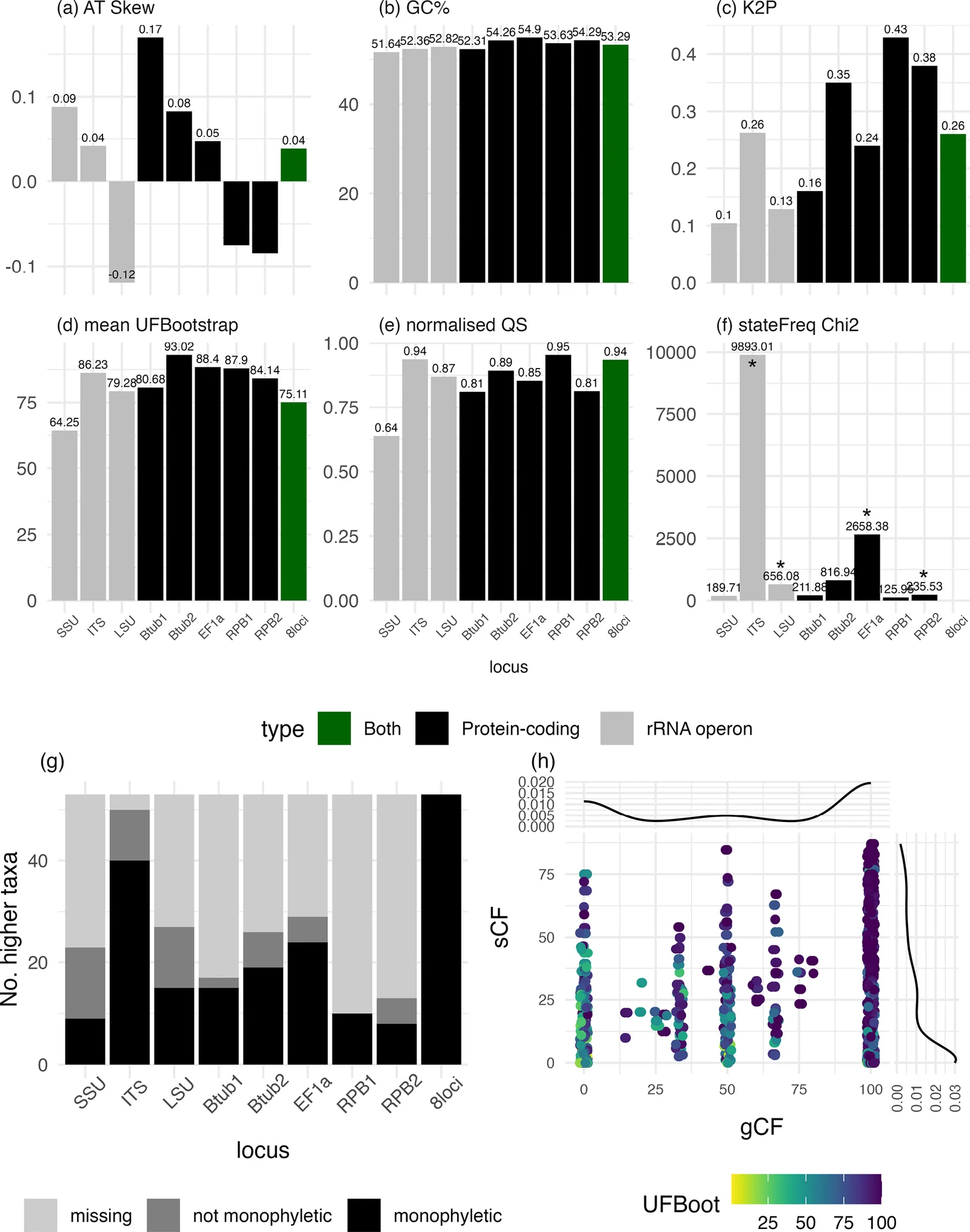
Plots comparing *Microascales* summary statistics of sequence alignments and phylogenetic trees for each locus and the concatenated eight-locus dataset. **a**. Alignment AT skew; **b**. Alignment GC%; **c**. Kimura 2-parameter (K2P) pairwise distance; **d**. Mean ultrafast bootstrap support; **e**. Normalised quartet support (QS); **f**. Nucleotide state frequency chi-square, with an asterisk indicating significance (*p* < 0.05); **g**. Number of higher taxa recovered as monophyletic (black), not monophyletic (dark grey), and missing (light grey); **h**. Scatter plot showing the relationship between IQ-TREE gene concordance factor (gCF), site concordance factor (sCF), and UFBootstrap. Each point represents one node in the concatenated species tree. Histograms show the distribution of concordance factors.

**Figure 2.**
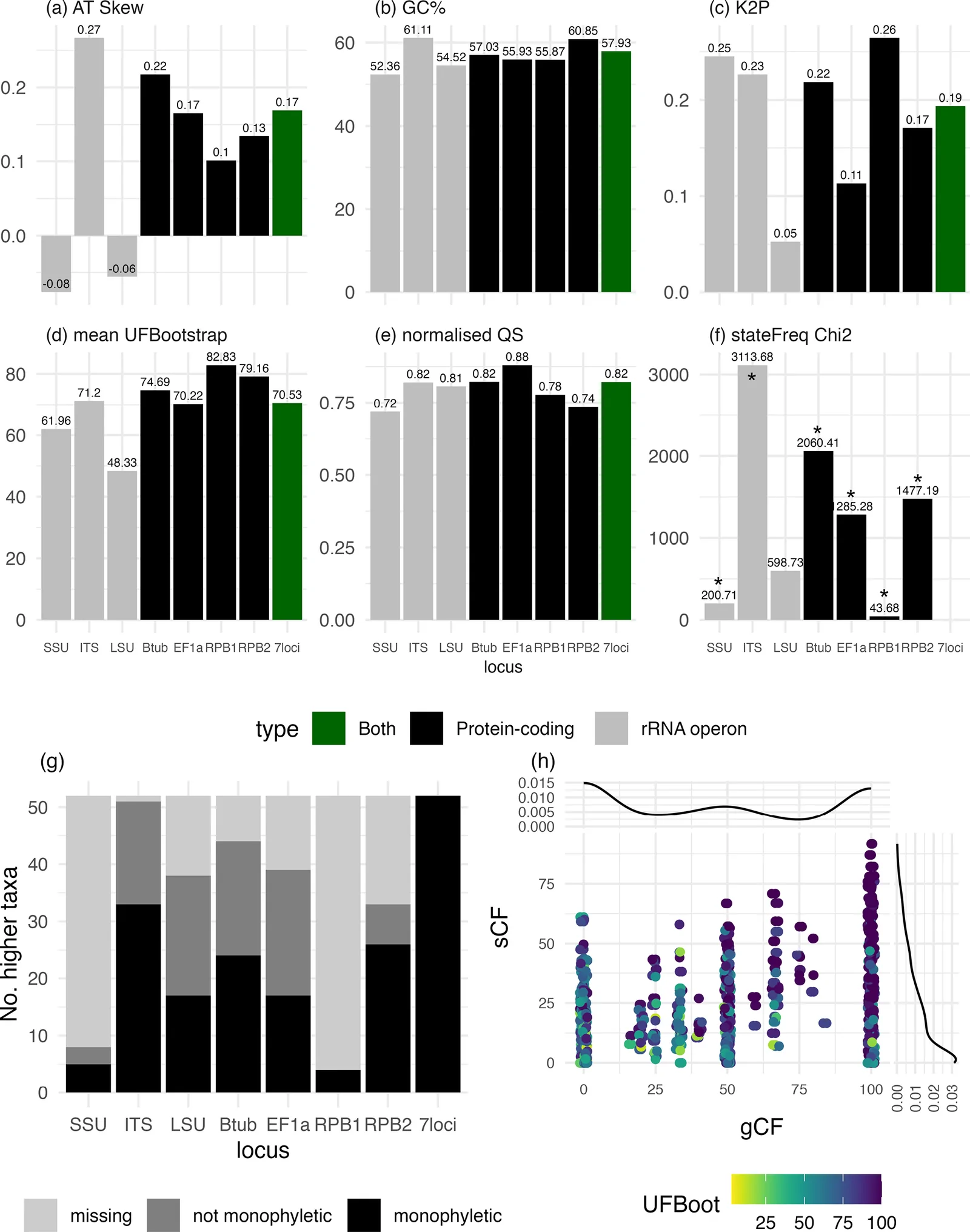
Plots comparing *Ophiostomatales* summary statistics of sequence alignments and phylogenetic trees for each locus and the concatenated seven-locus dataset. **a**. Alignment AT skew; **b**. Alignment GC%; **c**. Kimura 2-parameter (K2P) pairwise distance; **d**. Mean ultrafast bootstrap support; **e**. Normalised quartet support (QS); **f**. Nucleotide state frequency chi-square, with an asterisk indicating significance (*p* < 0.05); **g**. Number of higher taxa recovered as monophyletic (black), not monophyletic (dark grey), and missing (light grey); **h**. Scatter plot showing the relationship between IQ-TREE gene concordance factor (gCF), site concordance factor (sCF), and UFBootstrap. Each point represents one node in the concatenated species tree. Histograms show the distribution of concordance factors.

Pronounced differences in composition bias and evolutionary rates between ribosomal and protein-coding loci were observed across both orders. In *Microascales*, SSU and ITS showed positive AT skews, while LSU showed a marked negative skew (Fig. 1a). Conversely, in *Ophiostomatales*, ITS showed a strong positive AT skew, whereas LSU and SSU displayed negative skews (Fig. 2a). GC% remained relatively stable across loci for both orders (Figs 1b, 2b). In both orders, RPB1 showed the highest Kimura 2-parameter (K2P) distance, with ITS showing similar values (~0.2), whereas other loci differed between orders (Figs 1c, 2c). A chi-square test of homogeneity of base frequencies showed that nucleotide state frequencies differed significantly between taxa for ITS, LSU, EF1α, and RPB2 in the *Microascales* and for all genes except LSU in the *Ophiostomatales* (*p* < 0.05) (Figs 1f, 2f). The high ITSK2P, coupled with extreme state frequency heterogeneity (Figs 1f, 2f), shows that while ITS accumulates substantial sequence variation, it may be prone to homoplasy or compositional bias that could complicate deep phylogenetic reconstruction.

Evaluation of gene tree-building performance using mean ultrafast bootstrap (UFBootstrap) and normalised quartet support (QS) revealed the advantages of concatenating single-locus data. Within the rRNA operon, ITS consistently yielded the highest UFBootstrap support and quartet support compared with SSU and LSU in both orders (Figs 1d, 2d). However, in *Microascales*, SSU performed worst, whilst in *Ophiostomatales*, LSU performed worst. Protein-coding genes generally showed higher mean support than ribosomal operon genes, although the best-performing genes varied between the two orders. In general, locus trees showed greater agreement with the species tree in the *Microascales* than in the *Ophiostomatales* (normalised QS = 0.94 and 0.82, respectively). Furthermore, individual-locus quartet support varied considerably relative to the species tree for *Microascales* but showed little variation for *Ophiostomatales* (Figs 1e, 2e).

The recovery of the described higher taxa from both species trees in the gene trees was then investigated. For the *Microascales*, ITS clearly supported the highest number of known higher taxa, whereas SSU and RPB2 supported the lowest (Fig. 1g). For the *Ophiostomatales*, although ITS still had the highest number of supported monophyletic taxa, the difference was less pronounced, with ITS, LSU, β-tubulin, and EF1α all showing similar proportions of supported and unsupported taxa (Fig. 2g). SSU and RPB1 performed poorly across both orders; however, RPB2 was markedly different, supporting the fewest higher taxa in *Microascales* but a number similar to that supported by ITS in *Ophiostomatales*. Suppl. material 2: tables S2, S3 provide a detailed list of each higher taxon and its support in each locus tree.

**Figure 3.**
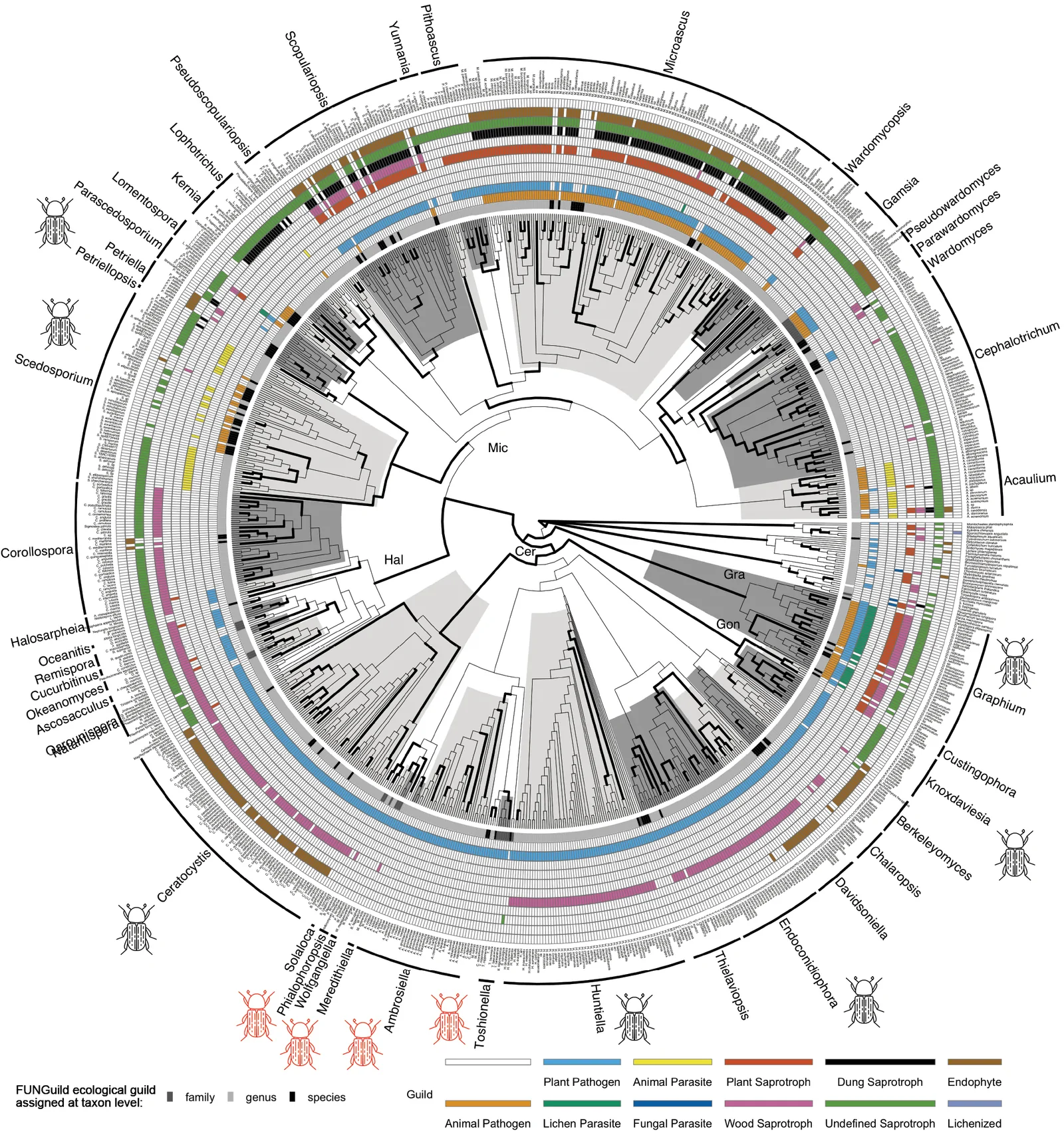
*Microascales*ML species tree reconstructed using the concatenated eight-locus dataset. Bold branches indicate UFBootstrap ≥ 95%. Higher taxa are labelled and highlighted by alternating light and dark grey blocks. Coloured tracks show ecological lifestyles assigned from FUNGuild. The inner track shows which taxon level was used to assign guilds. Note: A single higher taxon (genus or family) can have multiple assigned guilds, which may indicate different lifestyles across various species assigned to the taxon (see Discussion). Beetle icons indicate fungal taxa that associate with bark and ambrosia beetles. Red beetle icons indicate beetle-mediated fungal farming lineages, i.e., ambrosia fungi. The tree is shown as a cladogram for display purposes; see figshare Suppl. material 2: fig. S1 for branch lengths. Families are labelled as follows: Cer = *Ceratocystidaceae*, Gra = *Graphiaceae*, Hal = *Halosphaeriaceae*, Mic = *Microascaceae*.

Finally, site and gene concordance factors (sCF and gCF) were calculated to assess whether different alignment sites and genes support conflicting topologies. For most nodes in both species trees, the site concordance factor (sCF) was very low (Figs 1h, 2h, Suppl. material 2: figs S1, S2). Gene concordance factors (gCF) showed a split distribution, with many nodes receiving 100% gCF, whilst a smaller but still considerable number received very low or even 0% gCF (Figs 1h, 2h). In general, nodes with low gCF also tended to have low bootstrap support (Fig. 3h).

### Densely sampled species trees combined with lifestyle ecology data

In the *Microascales* concatenated ML species tree, the order *Microascales* was recovered as monophyletic (Fig. 3). At the family level, the subdivision of (*Ceratocystidaceae*, *Gondwanamycetaceae*) and (*Microascaceae*, *Halosphaeriaceae*), suggested by Réblová et al. (2011) and Bao et al. (2023). However, as in Bao et al. (2023), the placement of *Graphiaceae* within the order is unclear.

Within the monophyletic *Ceratocystidaceae*, the genera *Ceratocystis*, *Davidsoniella*, *Endoconidiophora*, *Meredithiella*, *Phialophoropsis*, *Solaloca*, *Wolfgangiella*, *Toshionella*, and *Ambrosiella* were recovered as monophyletic, with strong support (UFBootstrap ≥ 95%). *Gondwanamycetaceae* and both its genera, *Knoxdaviesia* and *Custingophora*, were strongly supported. The *Graphiaceae* and *Halosphaeriaceae* were both monophyletic and strongly supported. The *Halosphaeriaceae* genera *Oceanitis*, *Remispora*, and *Okeanomyces* were supported, but the remaining genera were not. The family also contained a high number of monospecific genera. The average taxonomic retention index (tRI) for the *Microascales* species tree was 0.6858.

The *Microascaceae*, though monophyletic, was not strongly supported. However, many genus-level clades received strong support: *Scedosporium*, *Petriellopsis*, *Parascedosporium*, *Lomentospora*, *Kernia*, *Lophotrichus*, *Pseudoscopulariopsis*, *Scopulariopsis*, *Yunnania*, *Pithoascus*, *Microascus*, *Wardomycopsis*, *Gamsia*, *Pseudowardomyces*, *Parawardomyces*, *Wardomyces*, and *Cephalotrichum*. Additionally, three large higher clades were strongly supported: *Scedosporium* and related genera, *Scopulariopsis* and related genera, and *Cephalotrichum* and related genera.

Fungal lifestyles/ecological guilds showed clear differences between families in the *Microascales* (Fig. 3). The *Ceratocystidaceae* were largely plant-associated as pathogens, endophytes, or saprotrophs. Most guilds were assigned at the genus level; hence, multiple guilds were reported for groups such as *Ceratocystis*. However, several well-known plant pathogens were assigned at the species level, e.g., *C. fimbriata*, *Thielaviopsis paradoxa*, and *Huntiella decorticans*.

The smaller *Gondwanamycetaceae* was also plant-associated, with both genera displaying two guilds: *Knoxdaviesia* as plant pathogens or saprotrophs and *Custingophora* as plant or wood saprotrophs. The *Graphiaceae* showed a higher diversity of lifestyles, with plant, animal, and lichen parasites/pathogens represented. Some key pathogens were also assigned at the species level, such as *Graphium penicilloides* on plants and *G. basitruncatum* on animals.

The *Microascaceae* also showed a diverse range of both plant- and animal-associated lifestyles, with a high turnover of lifestyles between sister clades. Compared with other families, they showed a prevalence of animal parasites/pathogenic species. These were mostly concentrated in the genera *Scedosporium*, *Lomentospora*, *Scopulariopsis*, and *Microascus*. The *Halosphaeriaceae* was largely saprotrophic on various substrates.

Bark beetle associations were not evenly distributed across the phylogeny. Many *Ceratocystidaceae* genera were reportedly bark beetle-associated (Fig. 3), as were genera in *Graphiaceae* and *Gondwanamycetaceae*. In contrast, no bark beetle associates were reported for the *Halosphaeriaceae*, and only one *Microascaceae* clade containing *Scedosporium* and *Petriella* was associated with bark beetles. Within the *Ceratocystidaceae*, bark beetle-associated genera were not monophyletic. The ambrosia lifestyle was reported in two clades within the *Ceratocystidaceae*, one containing *Ambrosiella* and *Toshionella* and a second containing *Phialophoropsis* and *Wolfgangiella* (Fig. 3).

**Figure 4.**
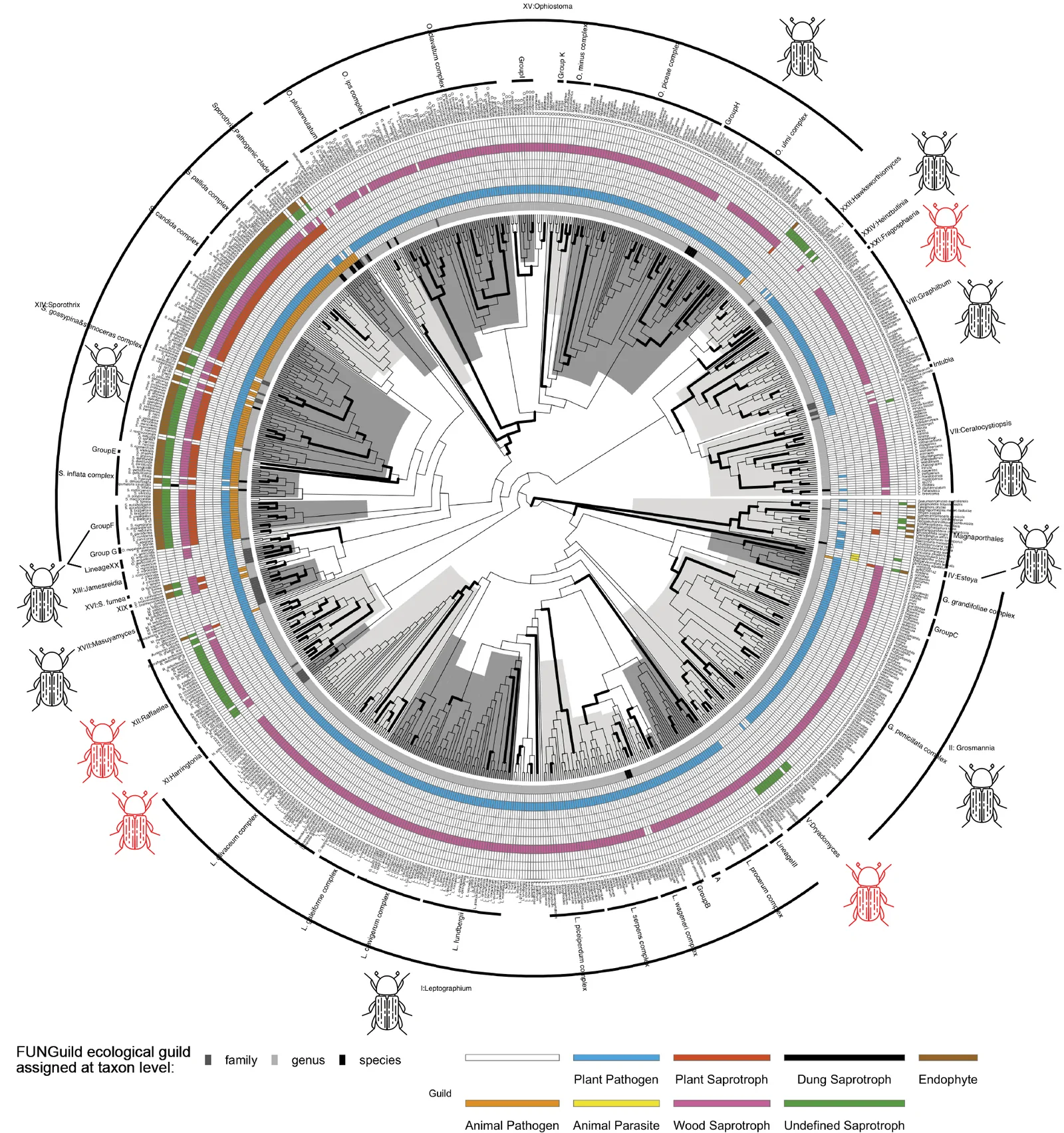
*Ophiostomatales*ML species tree reconstructed using the concatenated seven-locus dataset. Bold branches indicate UFBootstrap ≥ 95%. Higher taxa are labelled and highlighted by alternating light and dark grey blocks. Coloured tracks show ecological lifestyles assigned from FUNGuild. The inner track shows which taxon level was used to assign guilds. Note: A single higher taxon (genus or family) can have multiple assigned guilds, which may indicate different lifestyles across various species assigned to the taxon (see Discussion). Beetle icons indicate fungal taxa that associate with bark and ambrosia beetles. Red beetle icons indicate beetle-mediated fungal farming lineages, i.e., ambrosia fungi. The tree is shown as a cladogram for display purposes; see figshare Suppl. material 2: fig. S2 for branch lengths.

In the concatenated *Ophiostomatales* species tree, the order *Ophiostomatales* was recovered as a supported monophyletic group (Fig. 4). The results confirm most genera and many species complexes from the recent taxonomic revision of de Beer et al. (2022). Additionally, two large higher clades were recovered: (*Ophiostoma*, *Sporothrix*, *Hawksworthiomyces*, *Heinzbutinia*, *Fragosphaeria*, *Graphilbum*, *Intubia*, *Chrysosphaeria*, *Ceratocystiopsis*, *Jamesreidia*, *Masuyamyces*, *Raffaelea*, *Harringtonia*), here referred to as the *Ophiostoma*–*Sporothrix* clade, and (*Dryadomyces*, *Esteya*, *Leptographium*, *Grosmannia*), here referred to as the *Leptographium*–*Grosmannia* clade (Fig. 4). Both clades were supported by SH-aLRT (≥ 80%) but not by UFBootstrap. The position of the recently described *Jamesreidia*, *Masuyamyces*, and Lineage XX (*sensu*de Beer et al. (2022)) within the *Ophiostoma*–*Sporothrix* clade was also clarified for the first time. The average taxonomic retention index (tRI) for the *Ophiostomatales* species tree was 0.6931.

Within the *Leptographium*–*Grosmannia* clade, the genus *Esteya* was supported as monophyletic. *Grosmannia* was monophyletic and further subdivided into three species complexes: *G. penicillata*, *G. grandifoliae*, and Group C (*sensu*de Beer et al. (2022)). *Leptographium* was broadly supported, containing various supported species complexes (*L. serpens*, *L. piceiperdum*, *L. olivaceum*, Lineage III, and Group B *sensu*de Beer et al. (2022)). The *L. procerum*, *L. wageneri*, *L. lundbergii*, *L. clavigerum*, and *L. galeiforme* complexes were monophyletic but not supported by UFBootstrap.

Within the *Ophiostoma*–*Sporothrix* clade, the genera *Intubia*, *Graphilbum*, *Fragosphaeria*, *Heinzbutinia*, and Lineage XX were strongly supported. Despite lacking bootstrap support, the results resolve the recently described *Intubia* and *Chrysosphaeria* as a monophyletic group sister to *Ceratocystiopsis* in the *Ophiostoma*–*Sporothrix* clade, as seen in Nel et al. (2021). The large genus *Sporothrix* contained many monophyletic species complexes, although most were supported only by SH-aLRT. Within *Ophiostoma*, the *O. pluriannulatum*, *O. ips*, and Group K species complexes were strongly supported clades. The *O. clavatum*, Group I, *O. minus*, *O. piceae*, Group H, and *O. ulmi* complexes were also monophyletic but did not receive strong support.

The placement of *Graphilbum* has previously been unclear. The results resolve it as sister to the *Ophiostoma*–*Sporothrix* clade, Vanderpool et al. (2018) and the ML tree of Nel et al. (2021). This conflicts with other studies that resolve it as sister to the *Leptographium*–*Grosmannia* clade (de Beer et al. 2022 and the coalescent-based ASTRAL tree of Nel et al. 2021) or as sister to both groups together with *Aureovirgo*, as seen in Huang et al. (2025).

Fungal lifestyles were generally conserved across the *Ophiostomatales*, with most taxa listed as plant pathogens, wood saprotrophs, or both (Fig. 4). One genus, *Esteya*, was reported as an animal parasite. *Sporothrix* stood out compared with other genera, with examples of endophytes, undefined saprotrophs, plant saprotrophs, and animal pathogens that were largely absent from the rest of the order. Many important pathogens were assigned within ecologically variable genera. Within *Ophiostoma*, the *O. ulmi/ novo-ulmi* complex and *O. pluriannulatum* complex were reported as plant pathogens. Within *Sporothrix*, the well-known human pathogens were reported within the so-called ‘Pathogenic clade’ and the *S. inflata* complex (Fig. 4). The large genus *Leptographium* also contains known plant pathogens within the *L. wageneri* complex.

Beetle associations were also highly conserved, with almost all genera reportedly associated with bark and ambrosia beetles. Only the small genera *Hawksworthiomyces*, *Intubia*, *Chrysosphaeria*, *Jamesreidia*, and the *Sporothrix fumea incertae sedis* lineage were not reportedly beetle-associated. Additionally, all species complexes within *Ophiostoma*, *Leptographium*, and *Grosmannia* were beetle-associated. *Sporothrix* was a notable exception, with only the *S. gossypina*/* stenoceras* complex and Group F being beetle-associated. Ambrosia associations were reported in three distantly related clades: one clade with *Harringtonia* and *Raffaelea*, another with *Fragosphaeria*, and a third with *Dryadomyces*.

## Discussion

Understanding the evolutionary relationships of ophiostomatoid fungi remains a central challenge, given their highly convergent morphology, cryptic lifestyles, and the rapid expansion of sequence data. Rather than advancing isolated taxonomic descriptions or testing specific evolutionary ecology hypotheses, this study strategically assesses marker gene performance and provides a comprehensive, densely sampled multi-locus phylogenetic framework for the orders *Microascales* and *Ophiostomatales*. By integrating seven widely used markers across hundreds of species, this work synthesises fragmented literature and datasets into a single standardised framework, establishing a foundation for future functional, ecological, and biosecurity research. This supports a growing body of mycological literature demonstrating that separating data-rich reference frameworks from individual taxonomic monographs and trait evolution studies can be critical and valid endpoints in themselves (Kõljalg et al. 2005; Zhang et al. 2017; Põlme et al. 2020).

### Assessing locus performance, taxonomic coverage, and phylogenetic resolution for future systematics

A primary aim of this framework is the formal evaluation of marker gene performance across seven widely used fungal markers for both orders. The analysis reveals pronounced differences in composition bias, AT skew, and evolutionary rates between ribosomal operon components and protein-coding loci. Considering these data together reveals interesting patterns that may be missed when performance metrics are considered individually. For instance, whilst GC% is generally stable across loci, AT skew is markedly different, highlighting the importance of partitioning concatenated loci to avoid compositional differences violating substitution model assumptions. Additionally, whilst the internal transcribed spacer (ITS) remains the most densely sequenced region in public databases, it demonstrates extreme state frequency heterogeneity and high Kimura 2-parameter (K2P) distances in both orders. This indicates that although ITS accumulates substantial sequence variation suitable for species identification, its compositional bias and high evolutionary rate may obscure deep phylogenetic relationships.

Furthermore, evaluation using gene and site concordance factors (gCF/sCF) highlights a systemic lack of topological congruence across individual markers and alignment sites. One possible explanation for the broad distribution of low gCF scores across both trees is genuine evolutionary discordance, driven by variable selection pressures across loci. Tests for selection were not performed here, as indel-rich sequences such as the ribosomal operon are difficult to translate accurately, which is necessary for robust *dN/dS* analysis. Therefore, further tests on an expanded protein-coding dataset would be needed to demonstrate selection as the cause of the observed gCF distributions. Regardless, these statistics reveal the impact of gene incongruence that single-marker studies inherently fail to capture.

Combining ribosomal and protein-coding genes effectively dampens individual compositional biases, stabilising deep node support without losing the fine-scale resolution required at the species-complex level. Providing this diagnostic baseline is vital for preventing premature taxonomic changes based on single-gene anomalies and can help establish a clear quality-control standard for future fungal systematics (Stielow et al. 2015).

Combining broad taxon sampling (long data) with greater locus coverage (wide data) from genome-scale datasets is a key next step. Ophiostomatoid fungal genomes are becoming increasingly available, currently covering 76 *Microascales* species and 66 *Ophiostomatales* species (reference genomes from NCBI and JGI as of August 2026). Of these genomes, only two species are not covered by the multi-locus data (*Sporothrix thermara* and *S. variecibatus*). Therefore, although available genomic data are increasing, they are still at a much smaller scale than the almost 2,000-taxon dataset presented here. However, whilst genomes would not affect taxon coverage, they would certainly provide greater phylogenetic support, particularly at deeper nodes, either by producing a genome constraint tree or by filling in any missing marker genes for these taxa. Still, the fact that the topologies presented here successfully recover major backbone relationships from recent phylogenomic studies (Nel et al. 2021; de Beer et al. 2022; Huang et al. 2025) indicates that well-curated multi-locus datasets still carry strong phylogenetic information in the genomic era.

All sequences in this dataset originated from well-characterised, identified, and vouchered specimens or culture collections, ensuring traceability and enabling re-examination of morphological and ecological traits. Furthermore, many of the taxa covered may be difficult to resample for genomic data, making non-genomic datasets such as this invaluable for future studies. However, compiling and curating sequences into well-sampled, accurate alignments still required considerable manual curation and multiple rounds of tree reconstruction to correct all misidentified or misaligned sequences. This rigorous filtering is reflected in the almost tenfold decrease in sequences from the initial download to the final alignments. This drastic reduction is largely due to taxonomic redundancy from overrepresentation of a few heavily studied taxa, such as the phytopathogen *Ophiostoma novo-ulmi* or the human pathogen *Sporothrix brasiliensis*, which have large amounts of population-scale data. By providing a reproducible bioinformatics pipeline alongside lists of identified errors in sequence databases, this curated dataset helps prevent the amplification of database artefacts and redundancies in subsequent studies. This process is a necessary first step before generating new data, as it provides a summary of current knowledge that can act as a scaffold for selecting which lineages to sample and sequence for future genome-scale phylogenomic studies. This will ensure that genomes are evenly distributed across the phylogeny.

### Notes on ophiostomatoid fungal molecular systematics

Building on this methodological foundation, the results provide novel insights into ophiostomatoid fungal systematics. For example, the framework uncovers the stark difference in higher-taxon delimitation between *Microascales* families, where *Halosphaeriaceae* is dominated by many small or even monospecific genera, whereas species in its sister families are more evenly distributed across genera. Furthermore, genus support varies clearly between families, with most genera within the *Ceratocystidaceae*, *Gondwanamycetaceae*, and *Microascaceae* being monophyletic and strongly supported, whilst marine *Halosphaeriaceae* genera did not receive strong support despite being monophyletic.

In the *Ophiostomatales*, subsets of the two large clades seen in the species tree (*Ophiostoma*–*Sporothrix* and *Leptographium*–*Grosmannia*) are consistently recovered in phylogenetic analyses (Nel et al. 2021; de Beer et al. 2022; Huang et al. 2025) and support the idea that the *Ophiostomatales* may be better split into two smaller, more morphologically distinct families rather than the currently recognised single family, *Ophiostomataceae*. This was also suggested by de Beer et al. (2022), but the authors refrained from doing so because of weak support for deeper nodes in the phylogenetic tree.

By demonstrating precisely where single-locus markers disagree with the multi-locus concatenated tree, this study also helps reveal stable clades that can be relied upon for accurate identification, whilst highlighting unstable nodes such as *Graphilbum* and the *Intubia*–*Chrysosphaeria* lineage that may require targeted, genome-level sequencing to settle their placement.

### Evaluating macroecological trait data in ophiostomatoid fungi

By integrating the trees with large-scale databases such as FUNGuild, the ecological diversity of ophiostomatoid fungi begins to emerge. Contrasting patterns were observed between the two orders, with *Ophiostomatales* generally showing a lower diversity of lifestyles compared with the *Microascales*. The same pattern is seen with bark beetle associations, with *Ophiostomatales* being almost universally beetle-associated, whilst *Microascales* shows much greater variability. The absence of an aquatic-dominated *Halosphaeriaceae* analogue in the *Ophiostomatales* is particularly interesting, further emphasising the ecological diversity in the *Microascales*. Within the *Microascales*, bark beetle-associated genera are phylogenetically heterogeneous but not randomly drawn from across the whole tree; these genera are concentrated in the diverse *Ceratocystidaceae* and smaller *Graphiaceae* and *Gondwanamycetaceae*. However, even here, they are often sister to non-beetle associates.

Displaying beetle associations alongside FUNGuild data also emphasises the diverse pool of functional guilds from which beetles draw their fungal associates. For example, the results show that many beetle-associated taxa are also plant pathogens. This reflects a commonly reported mutually beneficial relationship that allows beetles and fungi to more effectively overcome host plant defences together (Ploetz et al. 2013; Joseph and Keyhani 2021; Jirošová et al. 2022). Retaining their capacity to digest plant compounds for nutrition whilst dispersing efficiently between plants via bark beetles also explains why these fungi can become devastating forest pathogens (Davis et al. 2019). Subsequent studies could therefore use this phylogenetic framework to directly test whether plant-pathogenic ophiostomatoid fungi are more likely to be associated with beetle vectors.

This dataset presents interesting evolutionary hypotheses for future testing. For example, whilst *Ophiostomatales* and *Microascales* form functionally similar beetle symbioses, previous research has shown that *Microascales* associations display greater plasticity, with many gains and losses (Mayers et al. 2015; Mayers et al. 2020a, 2020b). In contrast, the widespread beetle association seen in *Ophiostomatales* could reflect a more ancient beetle association gained by the common ancestor that was largely maintained and only secondarily lost in groups such as *Sporothrix*. Comparative ancestral state reconstruction of beetle associations for both orders could clarify this pattern.

The data also provide the basis for exploring the evolution of ambrosia fungi more broadly. Various studies have suggested that *Ceratocystidaceae* ambrosia lineages are more beetle host-specific compared with *Ophiostomataceae* ambrosia lineages (Skelton et al. 2019; Mayers et al. 2020a; Mayers et al. 2022; Osborn et al. 2023; Huang et al. 2025). If *Ophiostomatales* ambrosia fungi evolved within a context of pre-existing, widespread beetle association, they may have retained generalist traits, resulting in less host specificity. This difference has also been observed at the genomic level, with *Ophiostomatales* and *Microascales* showing different genomic adaptations to the ambrosial lifestyle, such as the expansion of different lineage-specific carbohydrate-active enzyme families (Huang et al. 2025).

However, the framework also highlights a key challenge in fungal macroecology. The observed difference in lifestyle diversity between the *Microascales* and *Ophiostomatales* is partly influenced by historical disparities in genus delimitation philosophies between the two orders. The fragmentation of *Microascales* into numerous small, restricted genera could artificially amplify apparent trait variability when mapped to broad trait databases, whereas the large, heterogeneous genera of *Ophiostomatales* could reduce apparent variability. Identifying this systematic bias is essential for users of macroecological databases (Hibbett et al. 2016). Therefore, whilst informative, further efforts are needed to populate databases such as FUNGuild with more species-level data to move beyond coarse genus-level categorisations.

The results reveal the limitations of linking ecological datasets such as FUNGuild to phylogenies for evolutionary analyses. FUNGuild compiles information from many sources and at different taxonomic and hierarchical levels, meaning that the resulting assignments are not always hierarchically consistent. For example, the genus *Ophiostoma* is listed as a plant pathogen or wood saprotroph, whilst *Ophiostomataceae* is listed solely as a plant pathogen. Additionally, genus-level assignments often show multiple possible guilds, whilst species-level assignments are predominantly single-state. It is therefore important not to interpret these assignments as indicating that taxa are ecologically polymorphic; rather, they reflect the way in which the information was compiled. Species-level records should therefore be treated as subsets or refinements of broader genus-level information rather than independent evolutionary character states. When these data are plotted onto phylogenies (Figs 3, 4), these inconsistencies are apparent and can suggest evolutionary transitions that are not necessarily supported by the underlying biology. The data also reveal a lack of uniformity in species-level trait data across ophiostomatoid fungi, indicating which clades require further investigation to characterise their ecology. Consequently, FUNGuild is most appropriately used to contextualise and summarise the current availability of ecological knowledge across the phylogeny rather than to infer trait evolution directly.

### Utility and applications of an ophiostomatoid fungi phylogenetic framework

Rather than being treated as a descriptive endpoint, these results provide a framework that can support several areas of future study:

#### 1. Accurate placement of environmental DNA metabarcodes through phylogenetic placement

Ophiostomatoid fungi are particularly difficult to isolate *in vitro* yet are frequently encountered as dark taxa in high-throughput beetle metabarcoding and environmental metabarcoding surveys (Miller et al. 2016; Ceballos-Escalera et al. 2025). However, public repositories either contain misidentified sequences and uncurated alignments or fail to cover the known species diversity of ophiostomatoid fungi, thereby preventing accurate ASV/OTU identification. With this resource, short environmental reads can now either be mapped onto a stable reference tree via evolutionary placement algorithms such as EPA-ng or pplacer (Matsen et al. 2010; Barbera et al. 2019) or be directly added to the alignments and included in new tree searches, both of which would substantially improve the accuracy of identifications in biodiversity monitoring.

Additionally, placing metabarcodes on reference phylogenies rather than reconstructing trees directly from short metabarcode regions allows phylogenetic diversity to be measured more reliably and enables analyses to move beyond species or OTU counts to identify field sites with unique or novel lineages. This can accelerate biodiversity exploration, especially in megadiverse or understudied regions, where sequence similarity-based approaches may struggle to confidently assign ophiostomatoid fungal sequences to known taxa. Moreover, by integrating different genetic markers into a unified phylogenetic framework, the same tree can be used to place sequences from different loci, an especially important feature for the *Ophiostomatales* genus *Raffaelea*, which is often difficult to amplify using universal ITS primers (Dreaden et al. 2014; Skelton et al. 2018).

#### 2. Filling phylogenetic gaps for macroevolutionary analyses

Time-calibration and trait evolution analyses, such as ancestral state reconstruction (ASR) via continuous-time Markov models, are highly sensitive to taxon sampling and topological stability (Heath et al. 2008a, 2008b). Conducting these analyses on sparse, unstable, or localised phylogenies can easily introduce errors into node age estimates and transition rate calculations. This framework establishes the necessary species-dense, robust topology that makes future macroevolutionary research mathematically rigorous and viable. Additionally, a better-sampled tree allows for greater and more accurate placement of fossils for time-calibration.

#### 3. Biosecurity and rapid diagnostics of emerging plant pathogens

Ophiostomatoid fungi include some of the most highly virulent vascular wilt pathogens responsible for catastrophic natural and human-managed forest declines (Brasier 1991; Wermelinger 2004; Ploetz et al. 2017). Coupled with the rapid range expansion of their bark beetle hosts, often through anthropogenic introductions, these fungi represent one of the largest current threats to global plant health. When novel beetle-fungal holobionts emerge in naïve forest ecosystems, plant health authorities rely on untargeted, resource-intensive mitigation measures such as large-scale slash-and-burn (Inward et al. 2024). By providing a stable reference phylogeny coupled with fungal lifestyle data, this framework will allow newly emerged strains to be placed and identified and enable a preliminary risk assessment of the pathogenic potential of fungal isolates based on their relationship to known pathogens. Combined with additional species- or strain-level ecological data, future projects could implement this phylogenetic approach to facilitate more targeted interventions.

## Conclusion

Ultimately, this study unifies the fragmented sequence landscape of ophiostomatoid fungi and demonstrates the value of broad and balanced taxon sampling founded on curated, voucher-derived sequences. By presenting a robust, open-access dataset alongside comprehensive locus performance metrics, this study provides the research community with a highly functional reference framework that can support future biodiversity discovery, evolutionary ecology, and biosecurity research.

## Additional information

### Artificial Intelligence (AI) use

The authors accept full responsibility for the content of the manuscript, including the disclosure of any use of AI.

No AI tools were used in the preparation of this manuscript.

### Author ORCIDs

T. Llewellyn https://orcid.org/0000-0002-8465-8031

A.P. Vogler https://orcid.org/0000-0002-2462-3718

### Data availability

The multiple sequence alignments and phylogenetic trees generated and analysed during the current study are deposited in the figshare repository: https://doi.org/10.6084/m9.figshare.30580976.

All bioinformatic code used to download and process sequences, build trees, calculate summary statistics, and parse FungalTraits data can be found at https://github.com/theo-llewellyn/Ophiostomatoid_Trees.

## Supplementary materials

Supplementary material 1 Ophiostomatoid sequences used in phylogenetic analyses and associated GenBank accession numbers Data type xlsx Explanation note The table also shows misidentified sequences and the reasons for their exclusion. https://binary.pensoft.net/file/1770264This dataset is made available under the Open Database License (http://opendatacommons.org/licenses/odbl/1.0/). The Open Database License (ODbL) is a license agreement intended to allow users to freely share, modify, and use this Dataset while maintaining this same freedom for others, provided that the original source and author(s) are credited. Theo Llewellyn, Alfried P. Vogler

Supplementary material 2 Supplementary data Data type docx Explanation note **table S2**. *Microascales* higher taxa supported by each locus in the eight-locus phylogenetic dataset. **table S3**. *Ophiostomatales* higher taxa supported by each locus in the seven-locus phylogenetic dataset. **fig. S1**. Phylogenetic tree of *Microascales* shown in Fig. 3, collapsed to the genus level, with node annotations showing UFBootstrap ≥ 95% (top left), SH-aLRT ≥ 80% (bottom left), gene concordance factor (top right), and site concordance factor (bottom right). **fig. S2**. Phylogenetic tree of *Ophiostomatales* shown in Fig. 4, collapsed to the genus level, with node annotations showing UFBootstrap ≥ 95% (top left), SH-aLRT ≥ 80% (bottom left), gene concordance factor (top right), and site concordance factor (bottom right). https://binary.pensoft.net/file/1770265This dataset is made available under the Open Database License (http://opendatacommons.org/licenses/odbl/1.0/). The Open Database License (ODbL) is a license agreement intended to allow users to freely share, modify, and use this Dataset while maintaining this same freedom for others, provided that the original source and author(s) are credited. Theo Llewellyn, Alfried P. Vogler
