# Supplementary material for: A robust phylogenetic framework for ophiostomatoid fungi: orders Ophiostomatales and Microascales (Sordariomycetes, Ascomycota)
Source: MycoKeys. 2026 Sep 11;140:103–29. doi: 10.3897/mycokeys.140.201772 (PMC13589300; doi:10.3897/mycokeys.140.201772)
Supplement: Supplementary file 1 [file mycokeys-140-103-s002.docx]

**Supplementary materials**

**Table S1.** Ophiostomatoid sequences used in phylogenetic analyses and associated GenBank accession numbers. The table also shows misidentified sequences and the reasons for their exclusion.

**Table S2.** *Microascales* higher taxa supported by each locus in the eight-locus phylogenetic dataset. Y = supported, N = not supported, and ‘-‘ = sequences for that taxon were not present for that locus.

|  | **SSU** | **ITS** | **LSU** | β**tub1** | β**tub2** | **EF1**α | **RPB1** | **RPB2** |
| --- | --- | --- | --- | --- | --- | --- | --- | --- |
| *Acaulium* | N | Y | Y | - | Y | Y | - | - |
| *Ambrosiella* | - | Y | N | Y | Y | Y | Y | - |
| *Ascosacculus* | - | Y | - | - | - | - | - | - |
| *Berkeleyomyces* | - | N | Y | Y | Y | Y | - | N |
| *Cephalotrichum* | N | Y | N | - | Y | Y | - | - |
| Ceratocystidaceae | Y | Y | Y | N | N | Y | Y | N |
| *Ceratocystis* | N | Y | N | N | Y | Y | - | Y |
| *Chalaropsis* | - | Y | N | Y | - | Y | - | - |
| *Corollospora* | Y | N | Y | - | N | Y | Y | N |
| Coronophorales | Y | N | Y | - | - | - | - | - |
| *Cucurbitinus* | - | Y | - | - | - | - | - | - |
| *Custingophora* | - | Y | - | - | - | - | - | Y |
| *Davidsoniella* | - | Y | N | Y | Y | N | - | - |
| *Endoconidiophora* | N | Y | N | Y | Y | Y | - | - |
| *Gamsia* | - | Y | - | - | Y | Y | - | - |
| Glomerellales | - | - | - | - | - | - | - | - |
| Gondwanamycetaceae | Y | Y | Y | - | - | - | - | Y |
| Graphiaceae | Y | Y | Y | Y | Y | Y | Y | - |
| *Graphium* | Y | Y | Y | Y | Y | Y | Y | - |
| *Halosarpheia* | - | - | - | - | - | Y | - | Y |
| Halosphaeriaceae | Y | N | Y | - | N | Y | Y | N |
| *Huntiella* | - | N | - | Y | - | N | - | - |
| *Kernia* | - | Y | - | - | Y | - | - | - |
| *Knoxdaviesia* | Y | Y | Y | - | - | - | - | - |
| *Lomentospora* | N | Y | - | - | Y | Y | - | - |
| *Lophotrichus* | - | Y | - | - | - | - | - | - |
| *Meredithiella* | - | - | - | Y | - | - | - | - |
| Microascaceae | N | N | Y | Y | N | N | Y | N |
| Microascales | N | N | N | - | - | - | - | - |
| *Microascus* | N | Y | N | - | N | Y | Y | Y |
| *Natantispora* | - | Y | Y | - | - | - | - | - |
| *Oceanitis* | - | Y | - | - | - | - | - | - |
| *Okeanomyces* | - | Y | - | - | - | - | - | - |
| *Parascedosporium* | - | Y | - | - | - | - | - | - |
| *Parawardomyces* | - | Y | - | - | Y | Y | - | - |
| *Petriella* | - | Y | Y | Y | Y | - | - | Y |
| *Petriellopsis* | - | Y | - | - | - | - | - | - |
| *Phialophoropsis* | - | Y | - | - | - | - | - | - |
| *Pithoascus* | - | Y | - | - | Y | N | - | - |
| *Pseudoscopulariopsis* | - | N | - | - | Y | Y | - | - |
| *Pseudowardomyces* | N | Y | - | - | - | Y | - | - |
| *Qarounispora* | - | Y | - | - | - | - | - | - |
| *Remispora* | - | Y | - | - | - | - | - | - |
| *Scedosporium* | N | Y | Y | Y | N | N | Y | Y |
| *Scopulariopsis* | N | N | N | - | N | Y | Y | Y |
| *Solaloca* | N | Y | N | - | - | - | - | - |
| *Thielaviopsis* | Y | Y | N | Y | - | Y | - | - |
| Torpedosporales | N | N | Y | - | - | - | - | - |
| *Toshionella* | - | Y | - | Y | - | Y | - | - |
| *Wardomyces* | - | Y | - | - | Y | Y | - | - |
| *Wardomycopsis* | - | Y | - | - | Y | Y | - | - |
| *Wolfgangiella* | - | Y | - | Y | - | - | - | - |
| *Yunnania* | N | Y | N | - | Y | - | - | - |

**Table S3.** *Ophiostomatales* higher taxa supported by each locus in the seven-locus phylogenetic dataset. Y = supported, N = not supported, and ‘-‘ = sequences for that taxon were not present for that locus. Higher-taxon names are formatted following de Beer et al. (2022).

|  | **SSU** | **ITS** | **LSU** | β**tub** | **EF1**α | **RPB1** | **RPB2** |
| --- | --- | --- | --- | --- | --- | --- | --- |
| *Ceratocystiopsis* | Y | N | Y | N | Y | - | Y |
| *Dryadomyces* | - | N | N | N | N | - | N |
| *Esteya* | - | Y | - | Y | - | - | - |
| *Fragosphaeria* | Y | Y | - | - | N | - | Y |
| *Grosmannia* | - | N | N | N | N | - | N |
| *G. grandifoliae* complex | - | Y | Y | Y | Y | - | - |
| *G. penicillata* complex | - | N | N | N | N | - | N |
| *Graphilbum* | - | N | N | N | N | - | Y |
| Group A | - | Y | N | Y | N | - | - |
| Group B | - | N | Y | N | - | - | - |
| Group C | - | Y | N | Y | Y | - | - |
| Group E | - | Y | - | - | - | - | - |
| Group F | - | N | N | N | N | - | - |
| Group G | - | N | - | N | - | - | - |
| Group H | - | Y | - | - | - | - | - |
| Group I | - | Y | Y | Y | Y | - | - |
| Group K | - | Y | - | - | - | - | - |
| Group XIX | - | Y | - | - | - | - | - |
| *Harringtonia* | - | Y | Y | Y | N | - | N |
| *Hawksworthiomyces* | - | N | N | N | N | - | N |
| *Heinzbutinia* | - | Y | - | Y | Y | - | Y |
| *Intubia* | - | Y | - | Y | - | - | - |
| *Jamesreidia* | - | Y | - | Y | Y | - | - |
| *Leptographium* | N | N | N | N | N | Y | N |
| *L. clavigerum* complex | - | Y | N | Y | Y | - | Y |
| *L. galeiforme* complex | - | Y | Y | N | Y | - | Y |
| *L. lundbergii* | - | N | N | N | Y | - | Y |
| *L. olivaceum* complex | - | N | N | N | N | - | Y |
| *L. piceiperdum* complex | - | Y | Y | Y | Y | - | Y |
| *L. procerum* complex | - | N | N | N | N | - | Y |
| *L. serpens* complex | - | N | N | Y | N | Y | Y |
| *L. wageneri* complex | - | N | N | Y | N | - | Y |
| *Lineage III* | - | Y | Y | Y | - | - | - |
| *Lineage XX* | - | Y | - | Y | - | - | - |
| *Magnaporthales* | Y | N | Y | - | - | - | - |
| *Masuyamyces* | - | Y | Y | N | Y | - | N |
| *Ophiostoma* | N | N | N | N | N | - | N |
| *O. clavatum* complex | - | N | N | N | N | - | Y |
| *O. ips* complex | - | Y | Y | Y | N | - | Y |
| *O. minus* complex | - | Y | Y | N | N | - | N |
| *O. piceae* complex | - | N | N | N | N | - | N |
| *O. pluriannulatum* complex | - | N | Y | N | Y | - | Y |
| *O. ulmi* complex | - | N | N | N | N | - | N |
| *Ophiostomatales* | Y | N | N | N | N | Y | N |
| *Raffaelea* | - | N | N | N | N | - | N |
| *Sporothrix* | Y | N | Y | N | N | Y | N |
| *S. candida* complex | - | Y | - | Y | Y | - | Y |
| *S. fumea* incertae sedis lineage | - | Y | - | - | - | - | - |
| *S. gossypina* and *stenoceras* complex | - | N | Y | N | N | - | Y |
| *S. inflata* complex | - | N | N | Y | N | - | Y |
| *S. pallida* complex | Y | N | N | N | Y | - | N |
| *Sporothrix* pathogenic clade | N | Y | Y | Y | Y | Y | Y |

**Figure S1.** Phylogenetic tree of *Microascales* shown in Fig. 3, collapsed to the genus level, with node annotations showing UFBootstrap ≥ 95% (top left), SH-aLRT ≥ 80% (bottom left), gene concordance factor (top right), and site concordance factor (bottom right). The tree is shown as a cladogram and was built with IQ-TREE using the eight-locus concatenated alignment. Families are labelled as follows: Cer = *Ceratocystidaceae*, Gra = *Graphiaceae*, Hal = *Halosphaeriaceae*, Mic = *Microascaceae*.

**Figure S2.** Phylogenetic tree of *Ophiostomatales* shown in Fig. 4, collapsed to the genus level, with node annotations showing UFBootstrap ≥ 95% (top left), SH-aLRT ≥ 80% (bottom left), gene concordance factor (top right), and site concordance factor (bottom right). The tree is shown as a cladogram and was built with IQ-TREE using the seven-locus concatenated alignment.
